# Germline TP53 and MSH6 mutations implicated in sporadic triple-negative breast cancer (TNBC): a preliminary study

**DOI:** 10.1186/s40246-018-0186-y

**Published:** 2019-01-10

**Authors:** Dandan Yi, Lei Xu, Jiaqi Luo, Xiaobin You, Tao Huang, Yi Zi, Xiaoting Li, Ru Wang, Zaixuan Zhong, Xiaoqiao Tang, Ang Li, Yujian Shi, Jianmei Rao, Yifen Zhang, Jianfeng Sang

**Affiliations:** 1 Department of General Surgery, Nanjing Drum Tower Hospital, Nanjing, Jiangsu Province China; 2 Department of General Surgery, Drum Tower Clinical Medical College of Nanjing Medical University, Nanjing, Jiangsu Province China; 3 Top Gene Tech (Guangzhou) Co., Ltd., Guangzhou, Guangdong Province China; 4 Department of Thyroid and Breast, Lianyungang First People’s Hospital, Lianyungang, Jiangsu Province China; 5 Department of Pathology, Affiliated Hospital of Nanjing University of Chinese Medicine, Jiangsu Province Hospital of Traditional Chinese Medicine, Nanjing, Jiangsu Province China

**Keywords:** TP53, MSH6, NF1, POLE, TNBC, Multi-gene testing

## Abstract

**Background:**

Germline BRCA1/2 prevalence is relatively low in sporadic triple-negative breast cancer (TNBC). We hypothesized that non-BRCA genes may also have significant germline contribution to Chinese sporadic TNBC, and the somatic mutational landscape of TNBC may vary between ethnic groups. We therefore conducted this study to investigate germline and somatic mutations in 43 cancer susceptibility genes in Chinese sporadic TNBC.

**Patients and methods:**

Sixty-six Chinese sporadic TNBC patients were enrolled in this study. Germline and tumor DNA of each patient were subjected to capture-based next-generation sequencing using a 43-gene panel. Standard bioinformatic analysis and variant classification were performed to identify deleterious/likely deleterious germline mutations and somatic mutations. Mutational analysis was conducted to identify significantly mutated genes.

**Results:**

Deleterious/likely deleterious germline mutations were identified in 27 (27/66, 40.9%) patients. Among the 27 patients, 9 (9/66, 13.6%) were TP53 carriers, 5 (5/66, 7.6%) were MSH6 carriers, and 5 (5/66, 7.6%) were BRCA1 carriers. Somatic mutations were identified in 64 (64/66, 97.0%) patients. TP53 somatic mutations occurred in most of the patients (45/66, 68.2%) and with highest mean allele frequency (28.1%), while NF1 and POLE were detected to have the highest mutation counts.

**Conclusions:**

Our results supported our hypotheses and suggested great potentials of TP53 and MSH6 as novel candidates for TNBC predisposition genes. The high frequency of somatic NF1 and POLE mutations in this study showed possibilities for clinical benefits from androgen-blockade therapies and immunotherapies in Chinese TNBC patients. Our study indicated necessity of multi-gene testing for TNBC prevention and treatment.

**Electronic supplementary material:**

The online version of this article (10.1186/s40246-018-0186-y) contains supplementary material, which is available to authorized users.

## Background

Triple-negative breast cancer (TNBC) has long been a focus of clinical concerns. It is defined by simultaneous lack of estrogen receptors (ER) and progesterone receptors (PR) and epithelial growth factor receptor 2 (HER2) expression; in other words, the growth of TNBC cells does not rely on hormone receptors and epithelial growth factors. This aggressive subtype accounts for 10–20% or more of all breast cancers depending on race and ethnicity (e.g., 19% in Chinese, 39% in Saudi Arabian) and is known to associate with early-onset of disease and poor prognosis [1]. However, although collectively classified as TNBC, the subtype is complicated with extreme heterogeneity revealed by expression/mutational profiling, genomic, and multi-omic studies [2–6]. Treatment options for TNBC are very limited due to lack of targeted therapeutics. Currently, the mainstream of treatment for TNBC still relies on chemotherapy. Despite high risk of developing chemo-resistance, TNBC has the highest response rate to neoadjuvant chemotherapy among all breast cancer types—an interesting phenomenon called “the TNBC paradox” [7]. Nevertheless, to achieve substantial improvement on the prognosis and survival of TNBC patients, new treatments targeting specific molecular defects are in urgent need.

Germline mutations in BRCA1/2 are intensively studied in breast cancers, and TNBC is highly related to BRCA1 germline mutations and family history. Prevalence of germline BRCA1 mutations varies in different race and ethnic groups, e.g., 24–30% in Ashkenazi Jewish, 7–8% in Chinese [1]. Roles of other predisposing genes in TNBC are less known. Some recent studies [8–10] indicated PALB2, FANCM, TP53, ATM, and RAD51D as potential candidates, but none of them have been comprehensively characterized in different race and ethnic groups, and yet, even in populations that have been studied, none of them exhibited prevalence comparable to BRCA1/2. It is well established that germline BRCA1/2 testing is highly recommended in TNBC with positive family history, but the clinical value of such a test in sporadic TNBC is still under debate, as the BRCA carrier probability is lower than 10% in sporadic TNBC patients < 60 years old [11]. Nevertheless, germline contributions to about 10% sporadic breast cancers are widely supported by research evidence [1, 3]. Therefore, the possibility exists that there are other candidate genes with prevalence equal or higher than BRCA1/2 in sporadic TNBC. This is thought to be more likely to happen in Chinese population, considering the relatively low BRCA1/2 prevalence in Chinese TNBC reported so far.

Unlike germline mutations which seem to concentrate on several particular genes, the somatic mutational landscape of TNBC is far more complicated and polymorphic. Comprehensive molecular characterization studies of breast cancer by The Cancer Genome Atlas (TCGA) revealed that TNBC exhibits a diverse mutational landscape with substantial similarity to that of serous ovarian cancers [3]. TP53 alteration is the hallmark of TNBC, with estimates that 60–80% of TNBC tissues harbor TP53 mutations [3, 4]. Hundreds of other genes and pathways have been shown to be altered with < 10% frequency, such as PIK3CA, PTEN, INPP4B, and MYC [3]. To our knowledge, somatic mutational profile of Chinese TNBC population is not clear so far. It is likely that Chinese TNBC possesses somatic mutational landscape distinct from that of TCGA, which is similar to the observation in lung cancers where EGFR mutations are much more frequent in Asians than in Europeans [12]. Studying somatic mutational landscape of Chinese TNBC may help identifying molecular targets more suitable for Chinese and Asians.

To address the hypotheses mentioned above, we conducted a preliminary study in both the germline and the somatic mutational landscapes drawn from 66 Chinese sporadic TNBC patients, based on a 43-gene panel. The results indicate that in Chinese sporadic TNBC, TP53 and MSH6 germline mutations might have comparable prevalence to BRCA1/2; for somatic mutations, NF1, POLE, ATM, and TP53 might be the most frequently mutated genes. Our preliminary study provides initial evidence of clinical values for testing and targeting non-BRCA genes in Chinese sporadic TNBC and serves as a foundation for further large-scale validation studies focusing on prevalence and clinical significance of non-BRCA genes.

## Methods

### Study cohort

A total of 82 sporadic TNBC patients were treated at Nanjing Drum Tower Hospital and Affiliated Hospital of Nanjing University of Chinese Medicine, Jiangsu Province Hospital of Traditional Chinese Medicine from January 2013 to May 2017. Sixteen cases lacking detailed clinical information and/or lacking enough materials for DNA extraction were excluded since the beginning of the study and for all the forthcoming experiments; the remained 66 cases were included in this study. Progression status of each patient was followed-up by January 2018. Each patient’s hormone receptors and HER2 status were determined by 2 independent pathologists using breast tumor tissue from biopsy or surgery. ER or PR negativity was defined as ≤ 1% of the tumor cells showing positive nuclear staining. HER2 negativity was defined as immunohistochemical staining score lower than 3. Peripheral blood and tumor tissue FFPE samples were collected from each patient. Note that for treatment decision purpose, each patient’s germline BRCA status was tested using BRCA1/2 panel followed by Sanger sequencing for confirmation of deleterious/likely deleterious mutations prior to this study. Detailed clinical characteristics of all patients extracted from medical records were listed in Table 1, which includes age at diagnosis (years), tumor stage (TNM), BRCA germline status (Sanger sequencing confirmed), cancer progression status, and treatment regimen. Informed written consent was obtained from each patient. This study was approved by the Ethics Committee of Nanjing Drum Tower Hospital, and all procedures performed within this study were done in accordance with the Chinese ethical standards and with the 2008 Helsinki declaration.

**Table 1:** Clinical characteristics of the studied cohort (*n* = 66)

Characteristics	Values (*n* = 66 patients)
Age at diagnosis (years)	
Median, range	51.5, 30–91
Stage	
I	13 (19.7%)
II	39 (59.1%)
III	12 (18.2%)
Undetermined	2 (3.0%)
BRCA germline status (Sanger sequencing confirmed)	
BRCA1 carrier	5 (7.6%)
BRCA2 carrier	2 (3.0%)
BRCA-negative	59 (89.4%)
Progression status	
PFS	53 (80.3%)
Relapse/metastasis	6 (9.1%)
Death	1 (1.5%)
Undetermined	6 (9.1%)
Whether (or not) treated with neoadjuvant therapy	
Yes	7 (10.6%)
No	55 (83.3%)
Undetermined	4 (6.1%)
Chemotherapy regimen	
TEC	16 (24.2%)
EC-T	20 (30.3%)
TC	3 (4.5%)
Other	10 (15.2%)
None	17 (25.8%)

*Abbreviations*: *PFS* progression-free survival, *TEC* docetaxel-epirubicin-cyclophosphamide, *EC-T* epirubicin-cyclophosphamide followed by docetaxel, *TC* docetaxel-cyclophosphamide

### Panel-based sequencing assay

All paired blood and tumor tissue FFPE samples were sent to TopGene Clinical Diagnostic Laboratory (Zhongshan, China) for next-generation sequencing using a capture-based method. Briefly, genomic DNA was extracted from each sample using Mag-bind blood and tissue DNA HDQ 96 kit (Omega Bioservices, Norcross, GA, USA) according to the manufacturer’s instructions. DNA quality was checked with Nanodrop (Thermo Fisher Scientific, Waltham, MA, USA). DNA quantification was performed with Qubit fluorometer 3.0 (Thermo Fisher Scientific, Waltham, MA, USA). Target sequences were captured from the extracted DNA using the custom panel (TopGene, China). PCR products were subjected to quality check with LabChip GX Touch24 (PerkinElmer). Pair-end sequencing was performed according to manufacturer’s protocols (Illumina, San Diego, CA, USA) using the NextSeq CN500 platform. The average depth of each sample was at least 300× and the read length was 2 × 150 bp.

### Bioinformatics analyses and variant classification

For each paired sample, reads generated from sequencing were subjected to reads processing and variant calling. Specifically, reads QC and filtering was performed with Fastp [13]; alignment of reads to human genome hg19/GRCh37 was performed using Burrows-Wheeler Aligner (BWA-mem, v0.7.15) [14]; GATK 3.6 toolkit [15] was used for local realignment around indels and base quality score recalibration. For germline variants, we used the GATK’s Haplotype Caller module for variant detection; for somatic variants, we used GTAK’s Mutect2 module and Lancet [16] for variant calling. Somatic variants with allele fraction < 1% were filtered to avoid false calls due to sequencing errors. VEP [17] and ANNOVAR [18] were used for variant annotation, and the identified variants were subjected to manual verification using Integrative Genomics Viewer [19]. Germline variants were then interpreted and classified by human experts according to the 2015 ACMG-AMP Guideline [20], with supporting information from pathogenicity prediction softwares, variant databases, and public literature.

### Mutation analyses

Somatic mutations of all patients were summarized to identify frequently mutated genes. Mutations of the 4 most frequently mutated genes were drawn for mutation spectrum plotting using the svg package (https://www.w3.org/Graphics/SVG/). To investigate affected pathways in TNBC, we divided the mutated genes into 3 classes: (1) homology directed repair pathway associated genes (HDR), (2) Lynch syndrome/colorectal cancer-associated genes (LS/CRC), (3) upstream master regulators (UPS) and generated heatmap of germline and somatic mutations of each patient with the pheatmap R package (https://CRAN.R-project.org/package=pheatmap). Driver gene analysis was performed using MutSig [21] and MuSiC2 [22] with a FDR (*q* value) threshold of 0.001. Significantly mutated genes identified concurrently by both softwares were considered as candidate driver genes.

## Results

### Patient characteristic overview

Clinical characteristics of the 66 sporadic TNBC patients were summarized in Table 1. Patients’ age of diagnosis ranged from 30 to 91, with a median of 51.5 years old. Germline BRCA1/2 status was determined prior to this study for treatment purpose (see the “Methods” section). Seven out of the 66 (10.6%) patients were germline BRCA1/2 carriers, confirmed with Sanger sequencing. Patients have undergone different treatment based on their genotypes and overall conditions, as listed in Table 1.

### Germline and somatic mutations

Using the 43-gene panel (Additional file 1: Table S1), a total of 39 germline deleterious/likely deleterious mutations were detected in 27 out of the 66 (40.9%) patients (Additional file 2: Table S2). Among the 27 patients, 8 carried two germline mutations and 2 patients carried three germline mutations. No recurrent germline pathogenic/likely pathogenic mutations were found. No significant differences on age of diagnosis, tumor sizes, stages, and prognostic status were found between germline carriers and non-carriers. Sixty-four out of 66 patients were detected carrying somatic mutations in genes within the panel.

### A comparison of germline and somatic mutational landscape

**Fig. 1:**
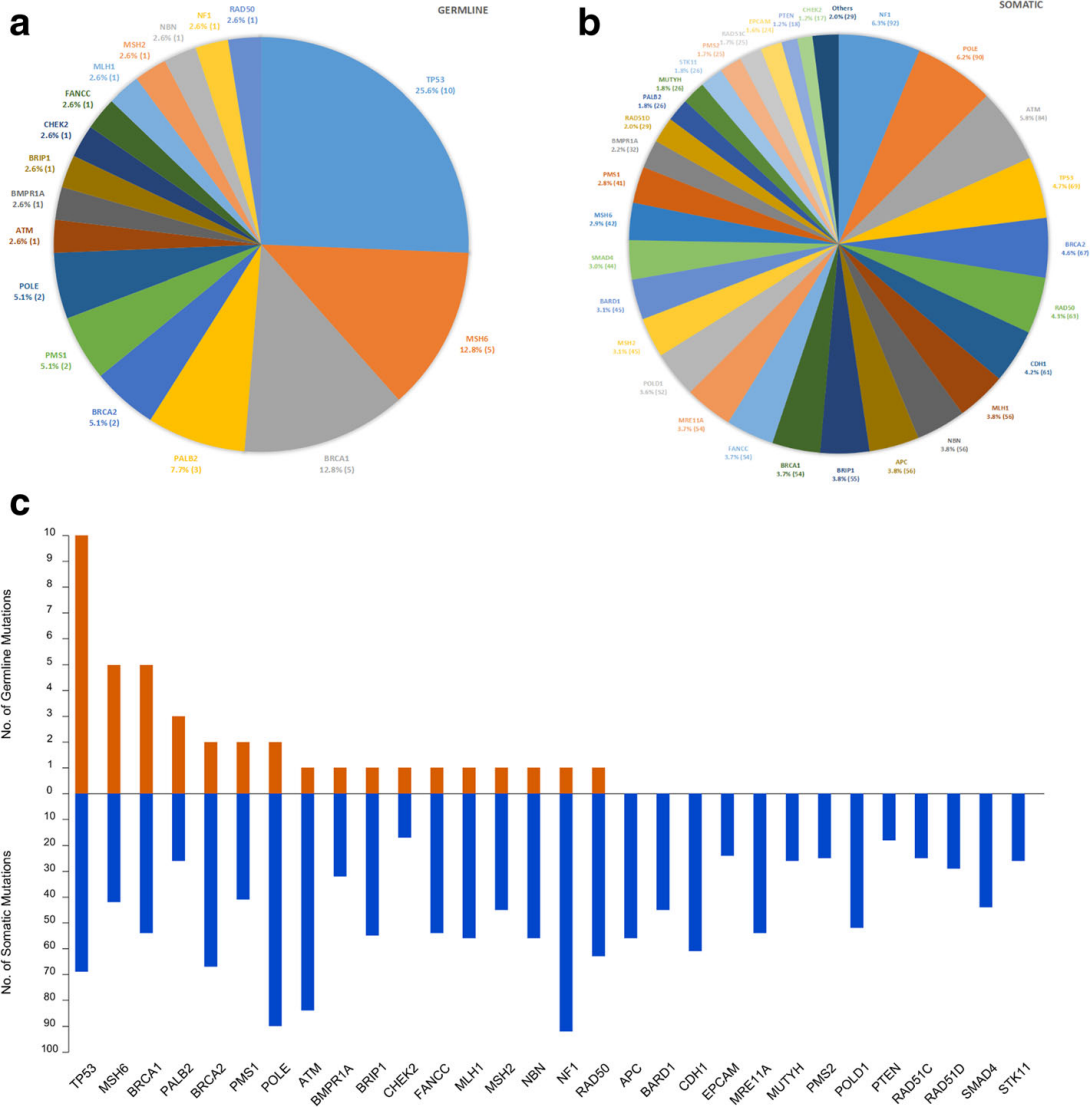
Germline/somatic mutational landscape of the studied cohort of sporadic TNBC patients. **a** Pie chart representing the proportion and number (in bracket) of deleterious/likely deleterious germline mutations identified within the 43-gene panel. **b** Pie chart representing the proportion and number (in bracket) of somatic mutations identified within the panel (those account for less than 1% of total mutation count were collectively grouped as “Others”). **c** Bar chart representing germline deleterious/likely deleterious mutation count and somatic mutation count of these genes

Deleterious/likely deleterious germline mutations were detected in 17 cancer susceptibility genes (Fig. 1a), whereas somatic mutations were detected in 33 cancer susceptibility genes (genes that account for less than 1% of total mutation count were grouped as “Others,” Fig. 1b).

Interestingly, TP53 and MSH6, rather than BRCA1, ranked the top for genes frequently harboring germline pathogenic/likely pathogenic mutations within the studied group of patients. The predicted contribution of germline susceptibility of these mutations were supported by multiple in silico prediction tools, ClinVar records and population frequency databases obtained from ANNOVAR (http://annovar.openbioinformatics.org/) and InterVar (http://wintervar.wglab.org/); all of these variants have a population frequency < 0.005 in the genome aggregation database (gnomAD, http://gnomad.broadinstitute.org/). Of the total 39 germline pathogenic/likely pathogenic mutations identified, TP53 made up more than a quarter (10 mutations in 9 patients), MSH6 and BRCA1 each took up 12.8% (each having 5 mutations in 5 patients). On the other hand, the somatic mutational landscape was much more complicated. Except TP53 which was also one of the top genes accounted for frequent somatic mutations, most germline susceptibility genes were relatively “less popular” in somatic mutations; instead, genes harboring very few or even no germline pathogenic/likely pathogenic mutations, such as NF1, POLE, ATM, and BRCA2, occupied bigger portions of somatic mutations (Fig. 1a, b, c). Despite TP53 did not yield the highest somatic mutation count, TP53 somatic mutations occurred in most of the patients and with highest mean allele frequency (45/66, 28.1%), followed by NF1 (37/66, 6.9%), POLE (35/66, 5.5%), and ATM (34/66, 4.5%).

### Somatic mutation spectra of the most frequently mutated genes

**Fig. 2:**
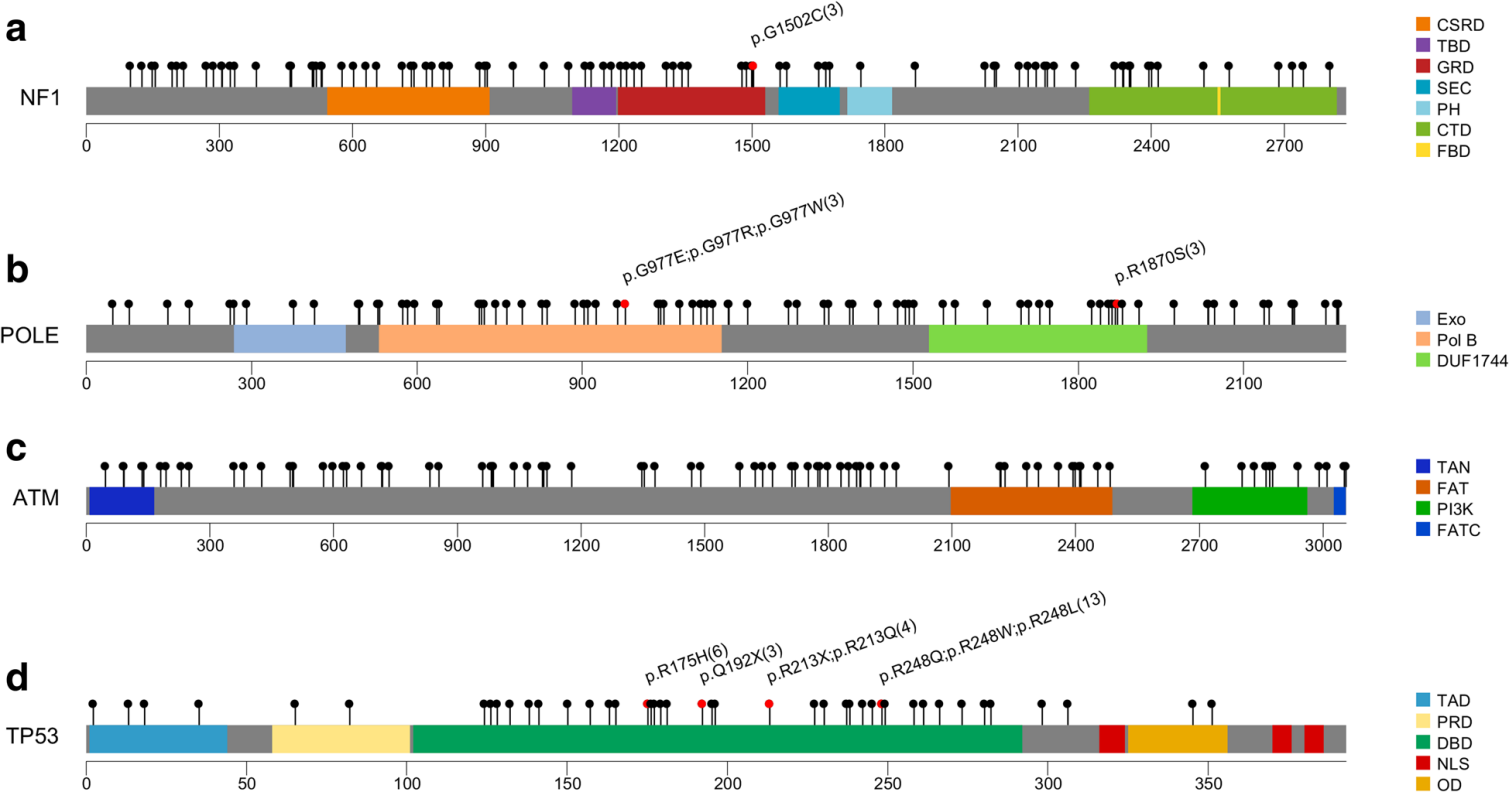
Lolliplot showing somatic mutation spectra throughout the whole protein sequences of the 4 most frequently mutated genes. Each scale bar represents the length (amino acids) of the protein sequence. Each lollipop represents a somatic mutation identified in this study. Positions which are recurrently mutated (total count ≥ 3) are highlighted with red lollipop and with text specifying the amino acid changes and number of occurrence. Functional domains are colored for each protein sequence. **a** Somatic mutation spectrum of NF1. CSRD cysteine-/serine-rich domain, TBD tubulin-binding domain, GRD GAP-related domain, SEC Sec14 homology-like region, PH pleckstrin homology-like domain, CTD C-terminal domain, FBD focal adhesion kinase-binding domain. **b** Somatic mutation spectrum of POLE. Exo 3′-5′ exonuclease domain, Pol B DNA polymerase type-B epsilon subfamily catalytic domain, DUF1744 domain of unknown function. **c** Somatic mutation spectrum of ATM. TAN telomere-length maintenance and DNA damage repair, FAT FAT domain, PI3K kinase catalytic domain, FATC FATC domain. **d** Somatic mutation spectrum of TP53. TAD trans-activation domain, PRD proline-rich domain, DBD DNA-binding domain, NLS nuclear localization signal, OD oligomerization domain

We computed the somatic mutation spectra of the 4 most frequently mutated genes: NF1, POLE, ATM, and TP53 (Fig. 2). Interestingly, mutations of NF1, POLE, and ATM seemed to distribute relatively even throughout the whole protein sequence, while mutations of TP53 concentrated on the DNA-binding domain (DBD). Moreover, some positions within DBD of the TP53 protein were concurrently mutated. For example, p.Arg175His was detected in 6 patients and Arg-248 was substituted by Gln/Trp/Leu in 13 patients. Nevertheless, the 10 germline pathogenic/likely pathogenic TP53 mutations identified were all positioned in the DBD region, but not overlapping with the somatic mutation hotspots.

### Affected pathways and significantly mutated genes

**Fig. 3:**
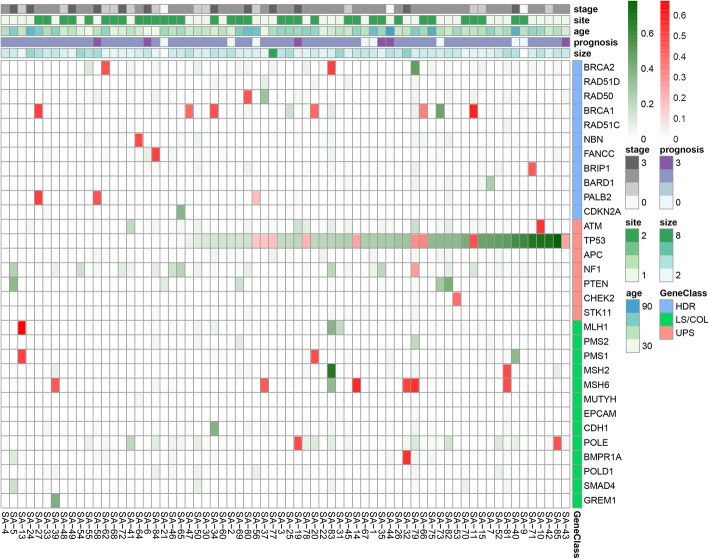
Heatmap representing germline and somatic mutated genes with their sum of mutation allele fraction (MAF) in each patient. Red boxes represent germline mutations, green boxes represent somatic mutations; intensity of color indicates the sum of MAF. If germline and somatic mutations co-occur in the same gene of the same patients, only germline mutations (red boxes) will be shown. Patients are ordered according to their sum of MAF for TP53 mutations. Clinical information (tumor stage, tumor site, age of diagnosis, prognosis, tumor size) of each patient was represented as sidebars. Stage: clinical stage of the tumor, 0 - unknown; 1 - stage I; 2 - stage II; 3 - stage III. Prognosis, 0 - death, 1 - unknown, 2 - survival until end of study; 3 - relapse/metastasis. Site: site of breast cancer, 1 - left, 2 - right. Size: size of tumor (cm). GeneClass: genes are divided into 3 gene classes according to their participation in different pathways, HDR homology-directed repair pathway-associated genes; LS/COL Lynch syndrome/colorectal cancer-associated genes; UPS upstream master regulators

To investigate the consequence of mutations in different pathways, we divided the mutated genes into 3 gene classes according to their roles in the affected pathways: (1) homology-directed repair pathway-associated genes (HDR), (2) Lynch syndrome/colorectal cancer-associated genes (LS/CRC), and (3) upstream master regulators (UPS). Figure 3 shows a heatmap of each gene’s sum of germline/somatic (red/green) mutant allele fraction (MAF) in each studied patient. As shown in Fig. 3, TP53 was altered in most of the patients with highest allele frequency, and mutations were most concentrated in upstream regulator molecules (UPS). Forty-six out of 66 (69.7%) patients were detected to carry mutations in all the three gene sub-classes, 13 patients (19.7%) were detected with mutations in two of the three sub-classes, and 7 patients (10.6%) detected with mutations in only one of the sub-classes.

Driver gene analysis was conducted using MutSig and MuSiC2. TP53 (376.9 mutations per Mbp, FDR < 10^−14^) and FANCC (219 mutations per Mbp, FDR < 10^−3^) were implicated as significantly mutated genes by both methods (Additional file 3: Table S3; Additional file 4: Table S4).

## Discussion

In this preliminary study, we comprehensively analyzed both germline and somatic mutations in 66 Chinese sporadic TNBC patients. The results supported our initial hypotheses, although they may not seem to be consistent with results done by many others. Instead of a BRCA1-dominant germline mutational landscape, we showed for the first time TP53 and MSH6 may be two strong candidates to have comparable prevalence to that of BRCA1. We also found that in TNBC somatic mutational landscape, besides TP53 which was commonly seen to be mutated, the high frequency of NF1, POLE, and ATM mutations were equally noteworthy.

One consistent finding of our result and others [3, 4] was the vital role of TP53 in tumorgenesis. In this study, TP53 was altered in 45 out of 66 (68.2%) patients with a mean allele frequency up to 28.1%. From a molecular point of view, the p53 tumor suppressor protein is the key controller of DNA damage-induced apoptosis. Inactivating mutations of TP53 may lead to anti-apoptosis and the accumulation of more deleterious mutations, which eventually results in unlimited proliferation and tumor development. Indeed, unlike other types of breast cancer, the growth of TNBC does not seem to rely on hormones or growth factors; however, it is the most fast-growing and relapse-prone subtype. It is not clear how early is the loss of p53 function event taken place during TNBC development [4], but it seems not surprising if chemo-resistance develops in TP53-mutant tumors, as their genomes are “guard-less” and never stop mutating—that the selection of drug-resistant mutations is only a matter of time. While somatic TP53 mutations in TNBC have been extensively characterized, the association between germline TP53 mutations and TNBC implicated in this study was previously not shown. Further validation studies are required to understand whether the cases found in this study are exception, or they reflect a Chinese-specific phenomenon. It is known that germline TP53 defect is associated with Li-Fraumeni Syndrome (LFS), a familial cancer predisposition disorder with very high cancer lifetime risk—73% for male and nearly 100% for female (mostly breast cancers) [23]. It would be interesting to study how similar/different are TP53+ sporadic TNBC- and LFS-related breast cancer in tumorgenesis.

Another interesting finding was the relatively high prevalence of MSH6 germline pathogenic/likely pathogenic mutations estimated from our study (5 mutations in 5 out of 66 patients, prevalence 7.6%) and the involvement of LS/CRC pathway in this TNBC cohort. This is much higher than the prevalence (5/8085) calculated from Sun et al. [10], which to our knowledge is currently the largest Chinese breast cancer study (a total of 8085 breast cancer cases, including 990 TNBC cases). In another multi-ethnic (European, African, Latin American/Caribbean, Asian) study including 35,000 women diagnosed with breast cancers, the prevalence of germline MSH6 pathogenic/likely pathogenic mutations is 2.2% in all breast cancers and 1.7% in the TNBC subgroup [24]. An estimated, although with ascertainment bias, germline MSH6 pathogenic/likely pathogenic mutation prevalence in general population from 50,000 women (most of which are assumed healthy subjects; multi-ethnic, Caucasian/European dominant) who had undergone hereditary cancer gene panel testing by GeneDx is about 0.3% (140/50000) [25]. The role of mismatch repair (MMR) genes such as MLH1, PMS2, MSH6, MSH2, and EPCAM are well established in Lynch syndrome-related tumors, such as colon, endometrial, and ovarian cancers. Association of germline MMR defects with breast cancers is less studied and without consensus conclusions. It is not until recently that the link between MSH6, PMS2, and breast cancers started to be comprehensively characterized [25]. Mechanistic studies probing the role of MSH6 and other MMR genes in breast cancer, and more particularly in the triple-negative subtype are awaited.

The somatic mutational landscape identified in this study (*n* = 66) was somehow different from published somatic mutational studies in TNBC, such as TCGA (*n* = 78) [3] and the Memorial Sloan Kettering Cancer Center (MSKCC) TNBC cohort (*n* = 39) [26]. The high TP53 somatic mutations were consistent in all the three studies, but the major difference lies in the high somatic mutation frequencies of NF1, POLE, and ATM in our Chinese TNBC cohort compared with the low frequencies in the other two. We came up with two hypotheses for the explanation of this inconsistency between cohorts: (1) the total sample size of all three studies were too small for a complete picture of the true somatic mutational landscape of TNBC; (2) there could be a true populational difference (by ethnicity or geographic locations) in TNBC somatic mutations. To answer these questions, comprehensive cancer gene panel or whole-exome studies with larger sample sizes and different population groups are required.

Our results of the somatic mutational landscape suggested potential therapeutic targets for Chinese TNBC. NF1 was frequently mutated in this study cohort (92 mutations in 37 patients). Interestingly, NF1 mutations are high in a TNBC subtype called “apocrine TNBC” with relatively high expression of androgen receptor (AR), although TNBC is overall lower in AR expression than other breast cancer subtypes [26, 27]. Pre-clinical studies of androgen-blockade have demonstrated benefits, and clinical trials of androgen-blockade-based combination therapies are currently underway [27]. Considering more than a half (37 in 66) TNBC patients in this study harbored NF1 somatic mutations, the population with potential benefits from androgen-blockade therapies could be large in China. POLE was another frequently mutated gene in our study (90 mutations in 35 patients). It is also a gene commonly mutated in CRCs. A common characteristic of POLE-mutated and/or MMR-deficient tumors is microsatellite instability (MSI) and hypermutation [28]. Hypermutation results in high tumor mutational burden (TMB) and consequently high tumor neoantigen burden (TNB), which renders higher chance of response to checkpoint blockade immunotherapies. Indeed, TNBC was shown to have higher TMB than other subtypes, and clinical trials of immune checkpoint inhibitors on TNBC are underway [29]. Considering the high frequency of POLE mutations (35 in 66 patients) shown in our study, immunotherapies could be beneficial to a significant portion of Chinese TNBC patients. Nevertheless, frequencies of somatic NF1 and POLE mutations in Chinese TNBC will need to be confirmed with further large-scale studies.

There are several limitations in this preliminary study. First, the 43-gene panel used in this study was designed in 2016. However, the recent few years have witnessed a great advance in understanding of the mutational spectrum of TNBC. Many candidate genes emerged to be related to this breast cancer subtype, such as AKT1, AKT3, INPP4B, and EGFR [1, 30]. Thus, an upgrade of the panel will be required for further comprehensive studies. Second, due to lack of public awareness of cancer diagnosis and treatment, we speculate that some patients enrolled in this study did not fully understand their family history. Cancer patients in the past and/or patients from less-developed area could die of cancer without correct diagnosis, so their family members may not know that cancer was the cause of death. Third, our study is a single-centered pilot study with a relatively small sample size. It is therefore difficult to obtain statistical significance, and the results may be more or less biased. As mentioned above, large-scale, multi-centered validation studies are definitely required to draw any conclusions.

## Conclusions

This is the first attempt of a comprehensive germline and somatic mutational analysis of Chinese sporadic TNBC using a multi-gene panel. Our data supported our initial hypotheses that some non-BRCA genes (such as TP53 and MSH6) might contribute to TNBC germline susceptibility as much as BRCA1/2 and that somatic mutational landscape of Chinese TNBC might differ from the one drawn from TCGA and other data. Our results suggested necessity of multi-gene testing for TNBC prevention and treatment.

## Additional files

Additional file 1: **Table S1.** The 43-gene panel used for this study. (PNG 42 kb)

Additional file 2: **Table S2.** Pathogenic and likely pathogenic mutations identified in the studied cohort. Annotations and classifications of each variant are provided as separated columns. (XLSX 13 kb)

Additional file 3: **Table S3.** Driver gene analysis by MutSig. The two genes with *q* value < 10^−3^ (TP53 and FANCC, highlighted in yellow) were considered significantly mutated. (XLSX 10 kb)

Additional file 4: **Table S4.** Significantly mutated gene test by MuSiC2. TP53 and FANCC were shown to have the highest number of mutations per Million base pair (Muts pMbp). (XLSX 12 kb)

## Abbreviations

AR: Androgen receptor
CRC: Colorectal cancer
DBD: DNA-binding domain
ER: Estrogen receptor
HDR: Homology-directed repair
LFS: Li-Fraumeni syndrome
LS: Lynch syndrome
MAF: Mutant allele fraction
MMR: Mismatch repair
MSI: Microsatellite instability
PR: Progesterone receptor
TCGA: The Cancer Genome Atlas
TMB: Tumor mutational burden
TNB: Tumor neoantigen burden
TNBC: Triple-negative breast cancer
UPS: Upstream master regulators
