# Supplementary figures and images for: Germline TP53 and MSH6 mutations implicated in sporadic triple-negative breast cancer (TNBC): a preliminary study
Source: Hum Genomics. 2019 Jan 10;13:4. doi: 10.1186/s40246-018-0186-y (PMC6327518; doi:10.1186/s40246-018-0186-y)

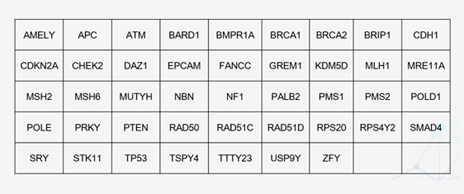

Supplement: Supplementary file 1 — Table S1. The 43-gene panel used for this study. (PNG 42 kb) [file 40246_2018_186_MOESM1_ESM.png]
